# The Case for Measuring Long Bone Hemodynamics With Near-Infrared Spectroscopy

**DOI:** 10.3389/fphys.2020.615977

**Published:** 2020-12-18

**Authors:** Chuan Zhang, Kevin K. McCully

**Affiliations:** Department of Kinesiology, University of Georgia, Athens, GA, United States

**Keywords:** metabolism, microvascular hemodynamics, optical spectroscopy, reactive hyperemia, oxygenation

## Abstract

Diseases and associated fragility of bone is an important medical issue. There is increasing evidence that bone health is related to blood flow and oxygen delivery. The development of non-invasive methods to evaluate bone blood flow and oxygen delivery promise to improve the detection and treatment of bone health in human. Near-infrared spectroscopy (NIRS) has been used to evaluate oxygen levels, blood flow, and metabolism in skeletal muscle and brain. While the limited penetration depth of NIRS restricts its application, NIRS studies have been performed on the medial aspect of the tibia and some other prominent bone sites. Two approaches using NIRS to evaluate bone health are discussed: (1) the rate of re-oxygenation of bone after a short bout of ischemia, and (2) the dynamics of oxygen levels during an intervention such as resistance exercise. Early studies have shown these approaches to have the potential to evaluate bone vascular health as well as the predicted efficacy of an intervention before changes in bone composition are detectable. Future studies are needed to fully develop and exploit the use of NIRS technology for the study of bone health.

## Introduction

Bone is a highly vascularized organ, and it has been well-documented that blood supply plays a key role in bone development (Marenzana and Arnett, 2013). Changes in bone blood supply has been observed in aging and pathological rodent models (Prisby et al., 2007; Stabley et al., 2015; Prisby, 2019). However, the assessment of bone vascular function in human has proven to be a difficult task, primarily because the stiff material properties of bone aren’t conducive to traditional measurement approaches. Positron emission tomography (PET) (Ashcroft et al., 1992; Frost et al., 2003) and dynamic contrast-enhanced magnetic resonance imaging (MRI) (Wang et al., 2009; Ma et al., 2013) are capable of measuring bone blood flow. However, these approaches have limited application due to the non-portable nature of this equipment as well as the high cost, high technical requirement to perform and analyze the PET and MRI scans. There is a growing need for non-invasive, easy to perform assessment for bone hemodynamics. Emerging research have suggested that near-infrared spectroscopy (NIRS) could potentially be a suitable tool for this mission (Binzoni and Spinelli, 2015; Meertens et al., 2018). However, little research has been performed using NIRS on bone, and many questions remain unanswered. Here we discuss some basic principles of NIRS as it can be applied to bone, summarize current studies that assessed long bone hemodynamics using NIRS, and point out promising directions for future research.

## Review of NIRS Technology

Portable NIRS devices utilize near-infrared light and detect changes of absorption (and scattering for some devices) at different wavelengths when penetrating biological tissues. A number of comprehensive reviews on NIRS technology have been published (Ferrari et al., 2011; Hamaoka et al., 2011; Quaresima et al., 2012; Jones et al., 2016; Barstow, 2019). Many comprehensive reviews have been published with details on the underlying physics as well as the application of the NIRS technique to the study of skeletal muscle (Hamaoka et al., 2007; Jones et al., 2016; Willingham and McCully, 2017; Hamaoka and McCully, 2019) and brain (Ferrari and Quaresima, 2012; Quaresima and Ferrari, 2019). Briefly, wavelengths in the near infrared region have biologically useful absorption characteristics. By resolving the light changes between light sources and detector through the use of the modified Beer-Lambert law, NIRS devices can determine concentrations of oxygenated (HbO_2_) and deoxygenated hemoglobin (HHb), and therefore measuring oxygenation in the tissue of interest (Jobsis, 1977). The determination of absolute (versus relative) concentrations of the heme species is a hotly debated topic, although even as relative changes, the measurements have been shown to have biological value. In skeletal muscle, myoglobin also contributes to the signal.

Near-infrared spectroscopy device emits photons from the light source, which travel through the biological tissue and are eventually partially picked up by a detector that’s usually located several centimeters away from the light source. During the penetration, there are many different scattering paths that the photons may potentially follow (Patterson and Pogue, 1994), with a portion of photons being absorbed and others being scattered. Only the photons that are scattered in such a way as to reach the location of the detector can be measured. In general, the depth of the penetration of the measured light is related to both the scattering and absorption coefficients of the medium, with greater depth found for low coefficient values. The penetration depth can also be modulated by changing the separation distance between the light source and detector. Greater separation distances result in greater penetration depths, with, however, less photons, returning to the detector (Patterson et al., 1995). Consequently, modern commercially available NIRS devices usually have separation distances between 2 and 5 cm. For less sophisticated NIRS devices, such as the continuous wavelength devices, no information regarding to changes of scattering when measuring the tissue is provided. Therefore, the concept of differential pathlength factor (DPF), which is the theoretical mean path length has been proposed and measured to quantitatively measure tissue optical properties. It should be noted that while there are existing studies to estimate DPF for brain and skeletal muscles (Ferrari et al., 1993; Duncan et al., 1995), such information is lacking for bone. On the other hand, more sophisticated NIRS such as the phase-modulated devices, can provide some information about the scattering coefficients and therefore allow for better quantitative evaluation of tissue optical properties. A question for future studies is whether the continuous wavelength devices are capable of providing useful measurements of oxygen levels in bone, or whether more expensive and less portable NIRS devices that provide scattering coefficients are required.

The application of NIRS to assess bone hemodynamics and oxygenation might be expected to be based on the rather extensive literature on the use of NIRS to study skeletal muscle in both healthy (van Beekvelt et al., 2001a,b; Ryan et al., 2013a,b; Southern et al., 2014; Zhang et al., 2020) as well as in clinical populations (Abozguia et al., 2008; Sjogaard et al., 2010; Bossie et al., 2017; Willingham et al., 2019). NIRS measurements of skeletal muscle can mainly be divided into three categories: (1) measuring levels of oxygen at rest and during exercise (Hesford et al., 2013; Niemeijer et al., 2017), (2) measuring the rate of re-oxygenation after ischemia or exercise (Willingham et al., 2016; Willingham and McCully, 2017; Lucero et al., 2018), and finally (3) using short periods of repeated ischemia to measure oxidative metabolism or mitochondrial capacity (Ryan et al., 2013b; Bossie et al., 2017; Sumner et al., 2020). NIRS measurements of oxygen levels in the brain have been used in a similar fashion to fMRI to measure activation of the brain (Duan et al., 2012; Heinzel et al., 2013). The question to be addressed is whether similar measurements can be performed on bone. One of the major challenges to the NIRS technique is the limited penetration depth of the light used, which is usually thought to be approximately half of the separation distance between the light source and detector (Miura et al., 2003). As a result, only surface tissues within 1–2 cm of the NIRS device can be evaluated. In addition, subcutaneous adipose tissue has been shown to affect the interpretation of NIRS signals from the deeper skeletal muscle (van Beekvelt et al., 2001a). These limitations pose major challenge for using NIRS on long bones, as they are usually located beneath skin, adipose tissue and various layers of skeletal muscles. As a result, few attempts have been made to utilize NIRS to assess long bone hemodynamics. On the other hand, there are a few anatomical locations of bone that are not covered with thick layers of adipose tissue and muscle. Tibial bone becomes the ideal candidate for NIRS studies, as the medial aspect of the tibia is generally free of most adipose or muscle tissues. Because of this, most studies of long bone using NIRS have focused on the medial aspect of the tibia (Binzoni et al., 2002, 2006; Draghici et al., 2018). In addition, some studies also looked at blood flow and hemodynamics at other prominent bone landmarks, such as the calcaneus (Pifferi et al., 2004; Sekar et al., 2015; Konugolu Venkata Sekar et al., 2016) and femoral head (Sekar et al., 2015; Konugolu Venkata Sekar et al., 2016).

## NIRS Assessed Long Bone Hemodynamics

### The Rate of Re-Oxygenation After Ischemia and Its Application in Bone

**FIGURE 1:**
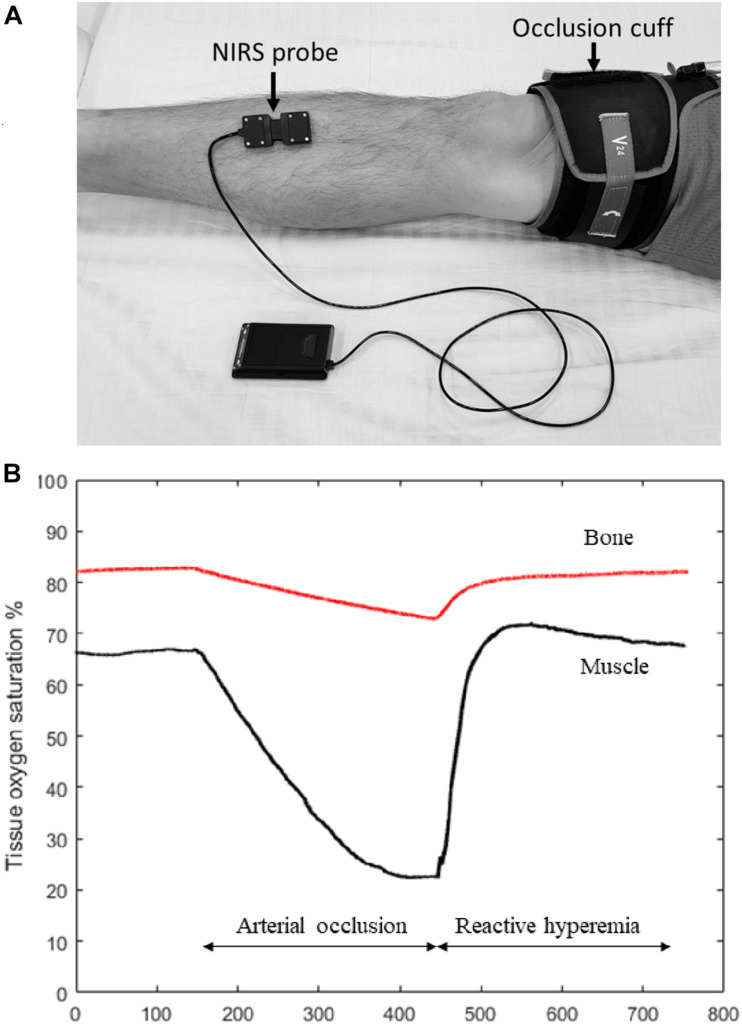
**(A)** Experiment set-up for measuring tibia reactive hyperemia. A NIRS probe is placed at the medial aspect of the tibia and attached to the skin with double-sided tape. Pre-wraps are also usually used to wrap around the NIRS probe to ensure that the probe is secure in place but also not too tight. An occlusion cuff connected to a rapid cuff inflation system is placed above the knee to provide arterial occlusion. **(B)** Figure showing muscle (black line) and bone (red line) NIRS oxygen saturation changes during the reactive hyperemia experiment. Compared to muscle, the magnitude of oxygen reduction in bone is expected to be smaller during arterial occlusion, and it takes longer time for bone to recovery oxygenation levels after the occlusion is released. The results in this figure are roughly based on results see in the few studies performed on tibial bone with the expected results from skeletal muscle.

Post-occlusive reactive hyperemia is a technique commonly used to assess peripheral hemodynamic responses (Willingham et al., 2016). This method usually involves placing the NIRS probe on the tissue of interest and placing a blood pressure cuff connected to a rapid inflation system above the joint (Figure 1A). The blood pressure cuff is rapidly inflated to 200–300 mm Hg (supra-systolic pressure), with the purpose to completely cut off the blood flow to the distal extremity. The ischemic occlusion usually lasts 3–5 min, and then the cuff is rapidly released. The NIRS device collects signal both during the ischemic occlusion and recovery period. With occlusion the HbO_2_ signal drops and the HHb signal rises in proportion to the metabolic rate of the tissue. As the cuff is released, the HbO_2_ and HHb signals return to their baseline levels, often showing an overshoot, and muscle oxygen saturation changes accordingly (Figure 1B, black line). There can also be a change in the HbO_2_ and HHb signals during the cuff and release periods due to changes in total blood volume in the tissue.

Using HbO_2_ signal from NIRS, a number of investigators were able to show that compared to healthy controls, individuals with peripheral arterial disease (PAD) showed significantly longer recovery time, slower rate of recovery, and lower maximal changes during reactive hyperemia (McCully et al., 1994, 1997; Kragelj et al., 2001). Importantly, the recovery of oxygen levels was correlated with ankle-brachial index (ABI), a clinical marker for disease severity in PAD. Overall, these studies demonstrate the feasibility of using NIRS to measure microvascular function as well as its application to clinical populations. In an attempt to find the most reproducible parameter to indicate vascular health during reactive hyperemia (Willingham et al., 2016) tested 20 healthy young individuals at two different time points for both a standardized as well as a leg elevation protocol (to simulate ischemic pre-condition). They concluded that time to half recovery of HbO_2_ signal after releasing the cuff was the most reliable parameter (Willingham et al., 2016).

Similar technique has been used to assess tibia hemodynamics. Binzoni et al. (2002) used NIRS to assess tibia HbO_2_ and HHb concentration changes following a 3-min ischemic occlusion on 13 healthy subjects with a wide range of age (25–72 years), and concluded that reperfusion rate decreases linearly with age starting from 30 years old. This study used the parameter of “Perfusion index,” which is the first derivative of the HHb-time recovery curve by mean of the Savitzky–Golay algorithm. This study is of great value in that it is the first study of its kind to demonstrate that NIRS signal is sensitive to changes in bone blood flow. In addition, the same study also measured muscle response during the reactive hyperemia, and was able to show that while muscle perfusion rate also declines with age, the absolute blood volume is greater in muscle than bone at any given age. However, due to the limited number of participants, the age associated results should be interpreted with caution. For example, in a study later on, the same group found that bone reperfusion capacity starts to decrease after 50 years old (Binzoni et al., 2003). Interestingly, this observation is consistent with the age commonly consider for major bone mass loss to start (Heaney et al., 2000). The discrepancy is likely the result of limited number of participants at a given age range as well as the small variations of perfusion rate in people with relative younger ages, and larger studies with more participants at specific age ranges are needed to determine the true onset age for bone perfusion rate to start decline.

Another study looked at tibial hemodynamic responses in people with spinal cord injury (SCI) using a 3-min occlusion protocol. It was found that individuals with SCI require longer time for tibia re-oxygenation compared to their able-bodied counterparts (Khakha et al., 2006). This study confirmed the feasibility of this technique on clinical populations. More recently and consistent with findings by Binzoni et al. (2002), a new study suggests that compared to muscle, tibia is characterized with slower desaturation rate and lower post-occlusive reactive hyperemic response as indicated by total oxygen index (HbO_2_ to total hemoglobin) as well as HHb changes in healthy population (Meertens et al., 2016). Moreover, it was shown that the intra-operator reliability for the NIRS measurements was high.

Based on previous studies, post-occlusive reactive hyperemia is a potentially useful method to study the hemodynamics of bone. Perhaps the most important issue related to post-occlusive hyperemia in bone is to link this measurement to bone health. This would assist the interpretation of impaired bone hemodynamic signals if they were identified. Other important new directions would be to confirm changes in NIRS based post-occlusive reactive hyperemia with established methods such as MRI. In addition, showing that the NIRS signals were sensitive to change and predicted changes in bone health will be important. However, many questions remain with regard to its application to measure long bone hemodynamics due to a lack of quality studies. Many NIRS derived markers were used to indicate hemodynamic response/vascular function, but it is unclear which one/ones are the best indicators. Studies aiming to identify markers that are longitudinally reproducible are needed. On the other hand, it may also be plausible to interpret HbO_2_ and HHb signals separately. In theory, during occlusion HHb increase in the tissue comes almost exclusively due to oxygen consumption, while changes in HbO_2_ signal may partially due to blood volume shift (Ryan et al., 2012), therefore HHb signal increase should reflect oxidative metabolism of the tissue. When the cuff is released, the HbO_2_ increase in the tissue should come almost exclusively from the new blood entering the tissue, so the change of HbO_2_ signal could reflect the blood flow influx. However, more research is needed confirm this inference.

### NIRS Measured Long Bone Oxygen Levels During Rest or Exercise

Near-infrared spectroscopy measured tissue oxygen levels has been used to indicate the balance between oxygen delivery and oxygen utilization, both at rest and during exercise (Hamaoka et al., 2011; Hesford et al., 2012). An early study on the tibia by Binzoni et al. (2006) showed that NIRS signal is sensitive enough to detect the hemodynamic changes in response to orthostatic stress changes caused by as little as 15° bed tilting in healthy individuals. Specifically, they were able to show that NIRS can detect the oxy-, deoxy- and total hemoglobin content increase due to the increased orthostatic pressure caused by bed tilting. However, another study found minimal changes to NIRS measured oxygen levels at four different body positions, including sitting, supine, 15° head down tilt (HDT), and 15° HDT plus lower body negative pressure (Siamwala et al., 2015). These studies were designed to improve our understanding on bone loss associated with spaceflight. More recently, Draghici et al. (2018) were able to demonstrate changes in oxygen levels in tibial bone during and after rowing. They found that while able-bodied individuals show clear increases in deoxygenated hemoglobin levels during and after rowing, individuals with SCI showed minimal changes (Draghici et al., 2018). These studies suggest that NIRS can measure changes in bone oxygenation during loading and exercise, and that such changes might indicated differences in response in clinical populations. Clearly more studies need to be conducted to better understand the changes in bone oxygen levels with loading and exercise, and how these changes might guide our understanding of bone health in clinical populations.

One clear distinction between muscle and bone hemodynamics as measured by NIRS is the magnitude of signal. Despite being a highly vascularized organ, when compared to muscle, the total amount of blood volume in bone is much less. This is demonstrated by the higher absolute heme signal and perfusion rate in muscle compared to bone (Binzoni et al., 2002; Klasing and Zange, 2003; Meertens et al., 2016). A potential implication for the blood volume disparity is the resting measurements. Due to the less total amount of hemoglobin in the bone vascular system, it is possible that the NIRS measured HbO_2_ and HHb as well as their derived measurements measured at rest on bone are likely to be less accurate than on muscle, especially for continuous wavelength NIRS devices which do not measure changing of scattering. On the positive side, changes in scattering are less likely to be an issue during hyperemic conditions, such as the post-occlusive reactive hyperemia or post-exercise recovery. However, studies designed to measure bone hemodynamics should take potential changes in scattering into consideration.

## Future Research Considerations

### NIRS Measurement Location Considerations

Near-infrared spectroscopy has been used to measure hemodynamics at various prominent bone sites. Several studies performed “optical biopsy” using broad-band time-resolved spectroscopy at human bony landmarks, including the calcaneus (Pifferi et al., 2004; Sekar et al., 2015; Konugolu Venkata Sekar et al., 2016), femoral head (Sekar et al., 2015; Konugolu Venkata Sekar et al., 2016), and some forearm locations (Sekar et al., 2015; Konugolu Venkata Sekar et al., 2016) where adipose and muscle are minimal. The advantage of this specialized NIRS technique is that in addition to quantifying HbO_2_ and HHb concentrations, they can provide more information to the measurement sites, such as water, lipid, and collagen concentrations. The disadvantages are the complexity of device set-up which could potentially prevent from being widely adopted. While a good reliability has been shown (Konugolu Venkata Sekar et al., 2016), it’s currently unknown how accurate these measurements are, and validation studies can be difficult to design due to a lack of means to compare to. Regardless, these studies showed the possibility of measuring bone hemodynamics at sites other than the tibia and are therefore of great value.

**FIGURE 2:**
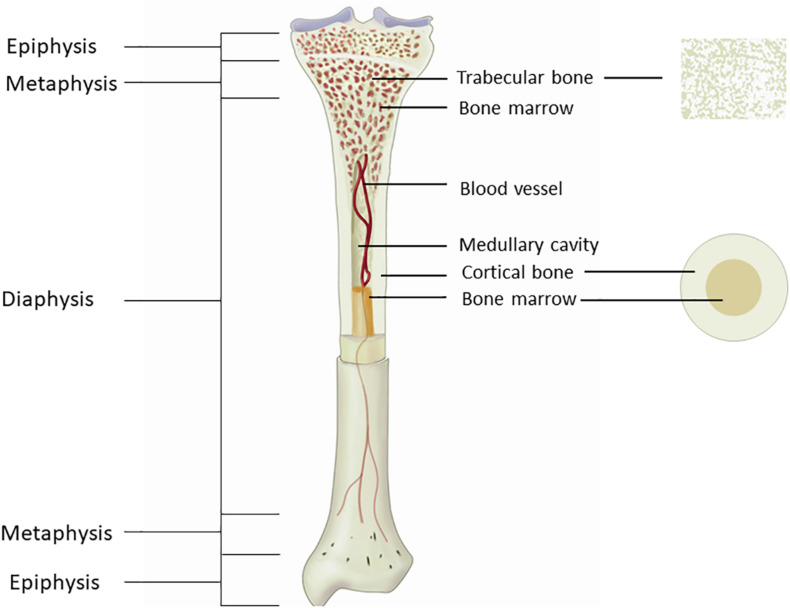
Figure showing the anatomy of the tibia. The long bone shaft is consisted primarily of cortical bone, with large medullary cavity inside for bone marrow to reside. Trabecular bone is presented primarily at the end of the bone, with bone marrow resides between the trabeculae space.

Most of the available studies that assessed hemodynamics on the tibia did not specify NIRS probe placement method. This is an important issue, because bone is not a homogeneous organ, and the composition of long bone varies greatly from the proximal to the distal end (Figure 2). The long bone shaft (diaphysis) is consisted primarily of cortical bone with large endosteal space. The hollow cavity is filled with bone marrow and is where most of vessels reside. As it moves along the bone to the distal and proximal ends, more trabecular bone is present, which gradually takes over the bone marrow space, and most blood vessels and bone marrow resides within the trabeculae space. Because long bone has different compositions at different segments, it is reasonable to assume that blood flow could be different as well. Therefore, it is very likely that the magnitude of NIRS signal measured at different segments of the tibia can vary. Whether placing the NIRS probe at different locations of the tibia can provide similar information of bone hemodynamic changes needs to be further explored.

### Relationship Between NIRS Measurements and Bone Development

Little information is known about the relationship between NIRS measured bone hemodynamics and bone development. Very recently, using the post-occlusive reactive hyperemia technique, it was shown that reduction rate in HbO_2_ at the tibia during arterial occlusion and subsequent recovery rate were correlated with areal bone mineral density (aBMD) measured by dual-energy X-ray absorptiometry (DXA) at the legs in healthy people (Meertens et al., 2020). This demonstrated the potential link between bone microvascular function and its development in human. Nevertheless, to date, studies examining the relationship between NIRS measurements and bone strength and microarchitecture are still lacking. Filling this gap in literature is of great importance, because DXA is a two-dimensional technology, and its derived aBMD measurement cannot truly represent the bone three-dimensional structure and strength. The value of adding bone strength and microarchitectural assessment in addition to BMD could help us differentiate those who do and do not fracture and improve our ability to predict fracture risks (Link et al., 1998; Majumdar et al., 1999; Ciarelli et al., 2000).

This topic is particularly important with respect to pathological conditions like type 2 diabetes (T2D). Adults with T2D usually present with normal or even high aBMD compared to similar aged healthy counterparts without T2D (Buysschaert et al., 1992; Akin et al., 2003; de Liefde et al., 2005; Oz et al., 2006). However, they have a higher fracture incidence rate (Nicodemus et al., 2001; de Liefde et al., 2005; Vestergaard, 2007). Human studies suggest that the trabecular bone density and structure are well preserved or even enhanced (Burghardt et al., 2010; Farr et al., 2014; Starr et al., 2018), whereas cortical bone properties are predominately compromised in T2D (Burghardt et al., 2010; Petit et al., 2010; Farr et al., 2014). Such disparity is intriguing, yet no conclusive explanations have been provided. It is possible that the high level of circulatory glucose causes microvascular damage to the bone through a mechanism similar to that of other peripheral vascular systems, which contributes to this paradox. Rodent models suggest that long-term T2D is associated with altered bone vascular function, and endothelium dependent-vasodilation is correlated with cortical and total volumetric BMD (Stabley et al., 2015). However, whether bone microvascular function changes can partly account for the trabecular and cortical bone development disparities in human T2D individuals has yet to be explored. The advancement of NIRS application on tibia hemodynamic assessment has enabled this possibility, therefore studies are urgently needed to address this gap in the literature.

Another physiological condition of interest is aging. Aging is a process accompanied with high bone fracture incidence rate (Ensrud, 2013) with declining bone mass and quality (Cohn et al., 1976; Nyssen-Behets et al., 1997; Agnusdei et al., 1998; Nalla et al., 2004; Koester et al., 2011). Animal studies have demonstrated that age-related deteriorations in the bone vascular system, which include but not limited to reduced blood flow, increased ossification, and decreased mass (Prisby et al., 2007; Prisby, 2014). Because bone remodeling is dependent on the supply of nutrients and other necessities by bone vessels (Prisby, 2019), alterations in the bone vascular function/blood flow could have tremendous negative impact on bone repair after microdamage, leading to bone fragility. To date, the role of bone vascular system deterioration on age-related bone compromise in human has not been examined. Establishing the relationship between these two could potentially provide us with a new therapeutic target for reducing the prevalence of osteoporosis.

### Implications for NIRS Measurements in Bone Intervention Studies

**FIGURE 3:**
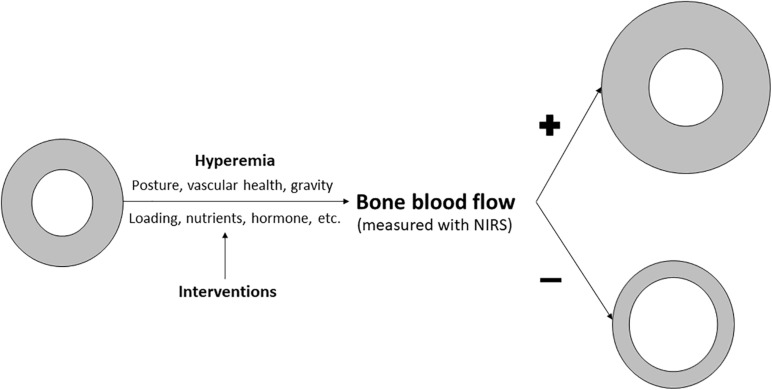
Figure showing how NIRS may be used to assess bone health and monitor intervention studies designed to enhance bone quality. Existing evidence suggest that posture, gravity, vascular health can impact bone blood flow. There is some evidence and a growing interest in bone hyperemia, as induced either by laboratory vascular occlusion procedure or exercise/physical activity, on bone blood flow changes primarily due to its modifiable nature. In addition, interventions designed for better loading, nutrition or hormonal conditions in human can enhanced bone blood flow. We propose that enhanced bone blood flow, which can be monitored by NIRS, will lead to greater total bone size and cortical (gray ring) expansion, resulting in stronger bones, whereas poor bone blood flow induced by a lack of these stimulation factors could lead to poor bone development or even bone loss, resulting in less cortical bone.

Although more research is needed, the evolvement of this field could have important clinical implications. Because bone is an organ considered to have slow turnover rate, interventions designed to improve bone density and quality and therefore reduce fracture risks generally takes many months, even years before the effects are large enough to be observed. On the other hand, improvement in bone microvascular function likely precedes the improvement of bone quality and strength (Figure 3). If a clear link between NIRS measured bone microvascular hemodynamics and bone strength and/or microarchitecture is established, it would provide us with another means to conduct early evaluation of the efficacy of the intervention program. However, more research is needed to confirm the notion that NIRS measured bone blood hemodynamic changes can serve as an early indicator of the effectiveness of the intervention programs.

### NIRS Measurement and Homeostasis

Other than the development of bone itself, it needs to be emphasized that bone is also important in maintaining proper homeostasis to human body. One of the key considerations of this aspect is the new red blood cells (RBC) generation. New RBC are formed almost exclusively at human bone marrow after birth. Impaired bone vascular function could have detrimental effects on the system RBC availability, as pointed out by a study suggesting that aging-related augmented bone marrow blood vessels ossification at femur corresponds with reduced RBC count in rats (Guderian et al., 2019). Vascular system in bone is responsible for its nutrient supply, waste exchange and RBC delivery to the system. Therefore, maintaining proper bone vascular health is not only important to bone, but could also have great implication on individual’s oxygen delivery system. Future studies are needed to determine whether NIRS derived bone measurements are related to human RBC count and subsequently, the aerobic capacity.

## Conclusion

In conclusion, existing evidence suggest that NIRS has great potential in assessing long bone blood flow and oxygenation. The portability, affordability nature of the NIRS testing equipment and the relatively easy data analysis procedure make it an ideal tool to be integrated as part of clinical or research assessment in evaluating bone health. However, the relative lack of studies has left many unanswered questions in this field. More studies are needed to evaluate and establish the most reliable testing and data processing procedure. More studies are also needed to establish the connection between NIRS measurements and bone strength and microarchitectural measurements, and determine whether they can be used to monitor changes due to intervention.

## Author Contributions

CZ and KM conceptualized the idea, performed the literature review, and wrote the manuscript. Both authors contributed to the article and approved the submitted version.

## Conflict of Interest

KM is the President of Infrared Rx, Inc., a NIRS software company. The remaining author declares that the research was conducted in the absence of any commercial or financial relationships that could be construed as a potential conflict of interest.
